# Key Contextual Factors Involved with Participation in Medical and Genomic Screening and Research for African American and Caucasian Americans: A Qualitative Inquiry American Journal of Community Genetics

**DOI:** 10.21203/rs.3.rs-4132207/v1

**Published:** 2024-03-26

**Authors:** Crystal Lederhos Smith, B. Connor Stark, McKenna Kobalter, Mary Carol Barks, Mariko Nakano-Okuno, Ellen Weger Romesburg, Nita Limdi, Thomas May

**Affiliations:** Washington State University; Washington State University; Washington State University; Washington State University; The University of Alabama at Birmingham Heersink School of Medicine; Washington State University; The University of Alabama at Birmingham Heersink School of Medicine; Washington State University

**Keywords:** genomics, genetic testing, medical research, racial disparities, medical trust

## Abstract

Tremendous progress has been made promoting diversity in recruitment for genomic research, yet challenges remain for several racial demographics. Research has cited intertwined fears of racial discrimination and medical mistrust as contributing factors. This study aimed to identify key factors to establishing trust in medical and genomic screening and research among African Americans and White Americans. Participants completed online focus groups and resulting transcripts were analyzed using a qualitative descriptive approach, with content analysis methods based on recommendations by Schreier. Fifteen African Americans and 23 Caucasian Americans participated in the study, 63% of which were female. The mean age of participants was 38.53 (SD = 16.6). The Overarching Theme of *Trust is Context Dependent* was identified, along with the following five themes describing elements influencing trustworthiness for our participants: 1) Professional Experience, Education, and Training Bolster Trust; 2) Trust Depends on Relationships; 3) Cross-checking Provided Information is Influential in Establishing Trust; 4) Trust is Undermined by Lack of Objectivity and Bias; and 5) Racism is an Embedded Concern and a Medical Trust Limiting Component for African Americans. To effectively address mistrust and promote recruitment of diverse participants, genomic research initiatives must be communicated in a manner that resonates with the specific diverse communities targeted. Our results suggest key factors influencing trust that should be attended to if we are to promote equity appropriately and respectfully by engaging diverse populations in genomic research.

## Introduction

Lack of diversity among biomedical research participants has been recognized as problematic for many years (Institute of Medicine. n.d.; Oh et al. 2015), and especially problematic for genomic research (Martin et al. 2019; Sirugo et al. 2019). In the U.S., historical attempts to address lack of diversity in biomedical research have centered around strengthened rules concerning informed consent and protection of an individual’s interests viz-a-viz “group” goods (e.g. scientific advancement) – concerns traditionally identified by studies of research participants (and thus reflective of the lack of diversity therein) (May et al. 2021). These attempts, while surely positive and necessary, have largely failed to adequately address issues of diversity, especially in genomic research (Popejoy & Fullerton. 2016). This failure is due to a variety of reasons we have explored elsewhere — many (if not most) of which center on perspectives of what inhibits participation that is skewed toward the concerns of society’s non-exploited and oppressed (May 2020; May et al. 2020, 2021). More recent attempts to address diversity in genomic research in particular have centered around protection of privacy, especially data security, as illustrated in the highlighted policies for “privacy and trust” and “data security” of the “All of Us” research program (https://allofus.nih.gov/about/all-us-research-program-protocol) (Mapes. 2020). While again both necessary and positive, these efforts continue to neglect essential elements of concerns that inhibit participation of historically exploited and/or oppressed groups in biomedical research: most importantly, concerns that the very nature of the research agenda itself poses a threat to that group’s equal treatment.

Historical exploitation based on race has been reflected in past scientific agendas like those manifest in the early 20th -century “Eugenics” movement, which attempted to correlate negative traits (low intelligence, criminality) to race despite far better socio-economic explanations (e.g. poverty) (Powell 2002; Reilly 2015). A more recent study of intelligence – “The Bell Curve” – similarly misapplies genetic explanations to phenomena better explained by historical disadvantages (e.g. access to education) to the detriment of African Americans (Herrstein & Murray 1996). Indeed, author Dorothy Roberts has published a detailed account of how artificial concepts of genetic race have perpetuated inequality in health (Roberts 2011).

Historical exploitation has extended beyond research to medical intervention itself. For example, the PHS Syphilis Study in Tuskegee (which denied access to available medical treatment) serves as a reminder of the disvalue placed on African American men throughout the 20th century; and the derivation of HeLa cells from an African American woman (Henrietta Lacks) without consent (or awareness) to develop cancer research that her own family has failed to benefit from, serves as an illustration of how research subjects can be exploited for larger societal benefits from which they are excluded. This has carried on to 21st century genomic medicine: in addition to lack of representation among genomic research participants, African American patients have proven far less likely to benefit from implementation of genomic medicine once translated to the clinic: for example, one study of 250 African American breast cancer patients found that 58.8% who met National Comprehensive Cancer Network genetic testing guidelines did not receive this as part of their care (Ademuyiwa et al 2019).

Elsewhere, author TM has argued that mistrust of the agenda of science is likely to be more prominent among members of historically exploited /oppressed groups, and must be accounted for if progress toward more representative diversity is to be made in biomedical (especially genomic) research (May 2020). Given the fact that historical exploitation has manifested not only in research but in medical intervention itself, addressing trust in the biomedical enterprise overall is essential for addressing health disparities across the board.

It is toward a deeper understanding of the factors influencing trust in biomedical science, and willingness to accept medical intervention, that we undertook this study. Given the fact that historical attempts to address lack of diversity through commonly accepted factors like consent and privacy have proven inadequate (as discussed above), our aim was to identify factors that might be overlooked by a scientific community that itself lacks truly diverse representation, particularly considering the greater prominence of those factors within historically exploited/oppressed groups. Our study focused on the most frequently identified group that lack representation (both in research participation and in clinical benefits derived from research): African Americans.

## Subjects and Methods

This study was conducted with adults aged 18 and older residing in the United States, who self-identified as African American or Non-Hispanic Caucasian. Recruitment was conducted through social media platforms and email contacts with volunteer collaborators across the United States. Interested participants completed an online interest form through REDCap data capture software (taking approximately two minutes). Qualifying participants were scheduled to complete a focus group using Zoom software. At the beginning of the focus group, participants completed consenting procedures, a short background survey, and entered their preferred email address for receipt of a $40 virtual gift card. Consenting, completion of the background information questionnaire, and participation in a focus group took participants approximately one hour. The incentive amount for participating in the focus group was intentionally selected to respect the value of participants’ time while avoiding a coercive inducement.

Focus groups were facilitated by research staff trained in qualitative interview methods. Please refer to Table 1 for the interview guide. Focus groups were recorded and uploaded to a HIPAA-compliant transcription site, wherein all identifying information was removed. A team of six of the manuscript authors participated in the qualitative analysis. Although the Washington State University Institutional Review Board determined that this study was exempt from review, informed consent was obtained from all individual participants included in the study. The authors affirm that the study was performed in accordance with the ethical standards as laid down in the 1964 Declaration of Helsinki and its later amendments.

We selected a qualitative descriptive approach (Sandelowski 2010; Schreier 2012), with content analysis methods based on recommendations by Schreier (2012), for the analysis of this data. Qualitative content analysis is a systematic, flexible data reduction method that involves combining concept-driven and data-driven analysis approaches to systematically describe the meaning of the rich qualitative material. The qualitative process included assigning successive pieces of information from the transcribed text to categories of our coding frame and then classifying these pieces according to categories and subcategories that we iteratively developed. An audit trail was kept throughout the process of analyzing the qualitative data to document thematic and coding decisions, promoting transparency and reliability. All researchers coded the qualitative data independently and then met to discuss, combine, and refine themes. Researchers met a total of 12 times to work through this iterative process. This analysis process allowed us to understand the most salient factors to establishing trust across African American and Caucasian American communities, including factors that increase and decrease skepticism of medical or research communication and information, as well as how engagement can be conducted to fully appreciate and address concerns that inhibit trust. It also allowed us to identify key sources of trusted information for each community, including discussion on the role of various community leaders and spokespersons, as well as the role of word-of-mouth, venues for dissemination of information, and effective messaging platforms (e.g., religious organizations, social media, television, radio, among others) for each community. Finally, this method allowed us to describe key contextual factors involved with participation in genomic screening and research. This was accomplished through an investigation of obstacles to participation and related race or racism concerns.

## Results

Thirty-eight people participated in the study, n = 15 African Americans and n = 23 Caucasian Americans. Approximately 55% (n = 23) of our sample identified as female and 5% (n = 2) identified as non-binary or transgender. The mean age of participants was 38.53 (SD = 16.6). For additional details on our sample see Table 2.

In response to a direct question designed to provide background for our study, we found that **Barriers to Participation in Genomic Screening Include Economics, Knowledge, and Trust**. This included economic or physical barriers; trust — or lack thereof — in the person collecting the information; concerns with privacy; validity of the intentions for use of the information; and lack of knowledge or understanding of the process, costs, and reason behind the testing.

*The main things that we talked about is trust in the organizations and the officials that are sending out this information to communities. I think those are—I think that’s definitely a big issue. I think political bias and mistrust from opposing political views can also be a big reason for mistrust, and then I think, honestly, one of the biggest things is just the lack of initial education on our different medical and health organizations, and I think a lot of that just comes from lack of information that is being taught in schools and other places too. Let people know what these resources and these organizations are for and the benefit that they provide to their communities.* (African American, participant 11 – 1)

*While it’s more accessible to many people, I still think that that can still be an economic barrier for those people that don’t have that extra cash*.” (Caucasian American, participant 1–1)

*In terms of genomic testing and that information in general, hindsight’s 20/20, but that’s a whole lot of personal information. It is a third party that has information related to my health. They may not be bound by the same rules. They might have the ability to be hacked by other personnel that can steal data pertaining to me. It could be sold to insurance companies to be used. It could be used as a screening method for allowance into certain insurances. I could imagine a future where Blue Cross Blue Shield has me do a genomic test to screen me for certain conditions before I am allowed to be on their insurance based on how they want to perceive me as a medical risk. I think the information manipulation is part of the genomic stuff that is still a little bit of a concern to me*. (Caucasian American, participant 1–2)

*I think that sometimes providers don’t explain everything they could explain, which of course is understandable because they don’t know what questions are in your head. I also think that there are time restrictions, how much time they can spend with the patient, et cetera, and so I think that sometimes you don’t get all the information that you could, you don’t have another opportunity.* (Caucasian American, participant 12 – 1)

Of these barriers, we focused on trust as this seems understudied and under accounted for in the context of our discussion above (Introduction). In this context, focus group participant responses to our direct questioning confirmed our view that trust is important for participation in genomic research and in uptake of clinical interventions.

Our analysis identified many components of trust and mistrust, applied by participants across medical providers, institutions, family, friends, and strangers (including media). Most saliently, the Overarching Theme of **Trust is Context-Dependent** arose. Categories that were identified prior to theme development were consistent across the African American and Caucasian American groups. However, one category (regarding racism) manifested differently across the groups and therefore developed into a different theme for each group. The following five themes identifying salient contextual elements that influence trustworthiness emerged in support of this conclusion: Theme 1) *Professional Experience, Education, and Training Bolster Trust*; Theme 2) *Trust Depends on Relationships*; Theme 3) *Cross-checking Provided Information is Influential in Establishing Trust*; Theme 4) *Trust is Undermined by Lack of Objectivity and Bias;* and Theme 5) *Racism is an Embedded Concern and a Medical Trust Limiting Component for African Americans*.

Overwhelmingly, both African American and Caucasian American groups reported their primary trusted source of medical information to be their medical provider, though most people also reported cross-checking information they received across multiple sources. Within and external to discussions of primary source of trusted information, factors impacting trust and mistrust came to light. The first theme identified was **Professional Experience, Education, and Training Bolster Trust.** Elucidation of this factor included conversations about the participants’ confidence in these factors and the strength of their impact on trust.

*I’m runnin’ under the assumption that this is their profession, and I’m gonna trust that they’re givin’ me the correct information, and I go from there.* (African American, participant 9 – 1)

*Medical professional…I go to the medical professional because that is a medical issue*. (African American, participant 4 – 1)

*General practitioner to specialists. […] They’ve been well-educated and have gone through rigorous training and have had specific standards and metrics that they need to meet that are well above the average person in order to then receive a license and then treat somebody*. (Caucasian American, participant 1–1)

*I would say my physician*. (Caucasian American, participant 1–4)

Conversations about relationships permeated the transcripts. Through these conversations we gathered information on how relationships impact trust from the point of finding and engaging with providers through how relationships might be leveraged in recruitment for its optimization. Participants explained that they had more trust in people closer (with stronger relationships) to them as they felt those closer people had a vested interest in their wellbeing. Discussions identified that trust through relationships is generally built over time through non-judgmental communication, responsiveness, listening, showing interest in the person, and validating their values or concerns. Open, consistent, non-judgmental communication and taking time, not rushing, and educating the patient during encounters impacted participants’ trust in their medical providers through the development of rapport. With this information gathered we identified theme two, **Trust Depends on Relationships**.

…*but a new provider, immediately, I would probably—I mean it would take a whole lot of time to build trust… You see these contradictions from the same profession. It really makes it hard for someone to really trust the system in terms of medical providers*. (African American, participant 4 – 2)

*I definitely would have to see them regularly and build that trust with them. […] I just know where that stems from with a lot of BIPOC folks, they have a lot of mistrust within the medical system. I think that would just start off with building that rapport and that equalness when it comes within—when it comes to the medical system. I think further on, the more trust that’s implemented, the easier it will get*. (African American, participant 9 – 3)

### *I would trust close friends, but I wouldn’t trust extended friends.* (Caucasian American, participant 1–2)

*I would think it’s just the rapport that I built up with them. It’s funny. My doctor just retired after forever and getting—the one who took her place, it’s not the same interaction. It’s not the same background. I think, even with my new doctor, even though she’s my doctor, I don’t trust her as much because I don’t have that rapport and I don’t have the history with her. It’s hard to—it’s building up that relationship*. (Caucasian American, participant 5 – 2)

Participants looked for information from/across multiple sources that was suggestive of consensus. This led to theme three, **Cross-checking Provided Information is Influential in Establishing Trust**. Such cross-checking can be achieved or represented in a variety of ways, including identifying existing consensus through online sources such as google reviews, or from referencing multiple sources (including medical professionals, social and other media, research literature, information provided by trusted medical sources such as the World Health Organization, or confirming stated facts with lived experience of others. Participants also found consensus through connecting with family and friends, religion and religious leaders, and information from political and community leaders.

*I’m the same—medical providers. A lot of times, I will get confirmation just with friends and family to kind of view with my symptoms or anything.* (African American, participant 9 – 2)

*I would say I get my medical information from the internet, but from a variety of sources, and I feel like, yeah, I’m always checking one site or one person against another rather than just pulling from one specific location*. (African American, participant 11 – 1)

*I would say I definitely trust medical professionals, but I also believe people generally speaking when they have experience. I don’t generally just believe one source. In other words, I might do some googling or some searching online. It’s not like I will believe just anything necessarily, I will look at different sources and see what the consensus is, if that makes sense*. (Caucasian American, focus participant 2 – 1)

*When it is something that I’m interested in or if I’m experiencing some sort of a condition, I will most likely do my own research and trust the literature that I’m looking at and the reviews that I’m doing in conjunction with my practitioner’s advice*. (Caucasian American, participant 1–1)

The fourth theme that arose was **Trust is Undermined by Lack of Objectivity and Bias**. This sometimes manifested as conflicts of interest, wherein discussion of political mistrust and hidden agendas arose. Discussions also suggested that trust can be undermined by perceptions that health professionals lack objectivity in one or several forms, including implicit bias, hidden agendas, or deriving undue conclusions from research studies.

*trust in the organizations and the officials that are sending out this information to communities. I think those are—I think that’s definitely a big issue. I think political bias and mistrust from opposing political views can also be a big reason for mistrust, and then I think, honestly, one of the biggest things is just the lack of initial education on our different medical and health organizations, and I think a lot of that just comes from lack of information that is being taught in schools and other places too. Let people know what these resources and these organizations are for and the benefit that they provide to their communities*. (African American, participant 11 – 1)

…*sometimes the doctors behind them—or above them, they have a different agenda, and they’re tryin’ to find out—put it this way. Experiment on different races and different people, things behind the scenes that we can’t comprehend. Sometimes, the doctors know, but they—it’s they’re just following orders. I say this because my daughter, she’s a scientist. I know a lot of things go on behind the closed doors, should I say.* (African American, participant 9 – 1)

*Any time that I see a celebrity promoting a certain drug or a device, I always question that. I always know that that’s coming from a biased perspective*. (Caucasian American, participant 1–1)

*Pharmaceutical companies as well in general. Obviously, they have a profit bias to make sure every drug does everything they want it to do with 95, 99 percent effective. Then also, yeah, anyone on TikTok or Instagram or whatever promoting any sort of new fad thing automatically just makes me roll my eyes, as well as just maybe the person who’s sharing that, also lowers my future things about whether or not I should—I can take their opinion on medical expertise. I’m much more skeptical of them in general as well. I think that, given social media, there’s been a lot more opportunity for grifters and other alternative medicines and—not alternative, but people even beyond the fringe of alternative medicine, but other things that are pure snake oil, I just absolutely do not, yeah, buy into that whatsoever. Anyone who says they’re doing some sort of cleanse or whatever because they saw it on TikTok or on Instagram, it’s just like, absolutely not*. (Caucasian American, participant 6 – 3)

Acknowledging that racism can be contextualized as a form of bias, we felt that the theme was unique and important to discuss individually. We intentionally conversed with participants about racism in the context of medical and institutional trust. Across these conversations, we saw divergence in the discussions. While our Caucasian American groups were cognizant of the presence of racism and how concerns about it could impact people’s behavior, including their own, they explained that they did not directly experience racism. No theme was developed for the Caucasian American groups. Our African American groups discussed racism across the transcripts, embedded consistently with conversations of its impacts on limiting trust. However, many (but not all) people in these groups stated that although they do not feel that they personally experience racism directly in their medical encounters, they are cognizant of institutional racism, such as devices not working as well on people with darker skin, and they are acutely knowledgeable of historical concerns. Many people expressed preference for a medical provider that matched their race. The result was our identification of a fifth theme, **Racism is an Embedded Concern and a Medical Trust Limiting Component for African Americans.**

*Well, okay, people without credentials, of course. Then I feel like it would depend in terms of specifics. For example, I would trust a black OBGYN way more than a non-black one…. Yeah, so me, as a black woman, and black women die the most from complications of childbirth, things like that in the US. I would just, I don’t know, I feel more—I feel safer with a black OBGYN than any other*. (African American, participant 15 – 1)

*I […] think some of it is systematic. Whether they weren’t taught about different bodies or they were given misinformation, not all of it is their fault. I know a lotta black and brown people, their symptoms get played down, whether they were—the doctor was taught something that was misinformed or whether they have some type of implicit bias.* (African American, participant 9 – 3)

*When we’re talking about racism, I’ve come to understand that it has brought about the absence of trust that health care providers and organizations, they genuinely care for their patients’ interest. It has made patients to trust—I mean to distrust their honesty. It has made patient to distrust them practicing confidentiality*. (African American, participant 4–4)

## Discussion

Trust is consistently identified as a critical factor influencing attitudes to genomics, as well as intentions to participate in genomic research (Lawler et al 2018; Nicol et al 2016). Results from our focus groups indicated that Trust is Context-Dependent and that, although there were many similarities across the conversations with our African-American and Caucasian-American groups, discussions of racism manifested differently across the groups, resulting is varying themes surrounding racism. Salient contextual elements influencing trustworthiness emerged, resulting in five themes: Theme 1) Professional Experience, Education, and Training Bolster Trust; Theme 2) Trust Depends on Relationships; Theme 3) Cross-checking Provided Information Is Influential in Establishing Trust; Theme 4) Trust Is Undermined by Lack of Objectivity and Bias; and Theme 5) Racism Is an Embedded Concern and a Medical Trust Limiting Component for African Americans.

We hypothesized that there would be greater variation in trusted sources of information between the African-American and Caucasian-American discussions based on preliminary evidence we had gathered in Alabama. It is quite possible that the starker differences between the groups that we saw in our preliminary work were related to the region in which we collected data. For this reason, our next steps will include use of the evidence gathered in this study to guide our development of an upcoming large Nationwide survey wherein we will examine regional differences in trusted sources of information.

This research is strengthened by its unique investigation of trusted sources of information, wherein the goal of identifying potential barriers and facilitators of genetic screening holds the potential to decrease inequity in participation. Although we took many steps to limit the impact of the limitations present in this work, it is important to acknowledge those limitations so the results can be appropriately considered. The first limitation of note is that people who have more distrust in genetic screening and medical providers and institutions may be less likely to participate in this type of research. In order to obtain input from these, more hesitant people, it will be important to engage with people they do trust, to facilitate communication and relationships and promote trust and engagement, as noted in the results of this manuscript. We have argued elsewhere that Community-Engaged Participatory Research is one potential strategy (May et al, 2021), but other strategies may also be effective. Further inquiry may identify additional or different trusted sources of information that are even more helpful in engaging hesitant groups.

To address mistrust and promote recruitment of diverse participants, genomic research initiatives such as “All of Us”, eMERGE, and others have placed priority on assuring privacy and promoting transparency (Precision Medicine Initiative 2022). To be effective, however, such principles must be communicated in a manner that resonates with the specific diverse communities targeted, and incorporate a recognition that mistrust of the scientific agenda itself may be as important or more important than privacy to historically exploited populations. The results from this research suggest key factors influencing trust in medical and genomic research that should be attended to if we are to appropriately and respectfully communicate about genomic research and engage importantly targeted populations to promote equity. Due to the qualitative method of inquiry used in this study, results from this research provide depth of knowledge and should be tested in larger samples in a quantitative manner before they are applied to large-scale recruitment efforts.

## Figures and Tables

**Table 1:** Interview Guide

· Who can you trust for information on medical interventions (for example, community leaders, spokespersons, friends, church leaders, medical providers, athletic coaches)?
○ Who would you NOT trust for information on medical interventions?
· What makes you feel like you can trust a medical intervention/institution/provider?
○ What makes you feel like you would NOT trust them?
· How trustworthy is word-of-mouth information related to medical interventions?
· What sources of information on medical interventions do you find most trustworthy?
· What sources of information on medical interventions do you find most biased?
· Where do you get your medical information?
○ Follow-up question: Do you believe this information is reliable and trustworthy?
· What contextual factors (e.g., history, location, community) impact your trust in medical interventions? Why?
· Are there specific concerns or worries regarding race or structural/institutional racism that inform your decisions to receive or to not receive medical interventions?
· What obstacles do you see to participation in genomic screening? [Potential prompts: psychological or physical barriers]
· Are there specific concerns or worries based on race or structural/institutional racism that inform your decision to participate or not participate in genomic screening?
· What sources of information on genomic screening are most trustworthy? (e.g., religious organizations, social media, television or radio, flyers, advertisements, direct communication)
○ What sources of information on genomic screening are LEAST trustworthy?
· How might recruitment for genomic screening initiatives or research protocols be better/more effectively implemented?
· What contextual factors (e.g., history, location, community) impact trust in genomic screening? Why?
· What concerns or obstacles have not been identified in this discussion that you think it would be important for us to know?

**Table 2.** Participant Demographics

	African American (n = 15)	Caucasian American (n = 23)	Total Sample (n = 38)
Age*	35.07 (17.9)	40.78 (15.6)	38.53 (16.6)
Gender			
Man	5 (33.3%)	8 (34.8%)	13 (31.0%)
Woman	8 (53.3%)	15 (65.2%)	23 (54.8%)
Non-binary	1 (6.7%)	0	1 (2.4%)
Transgender	1 (6.7%)	0	1 (2.4%)
Marital Status			
Single	9 (60%)	6 (26.1%)	15 (35.7%)
Married or Cohabitating	6 (40%)	14 (60.9%)	20 (47.6%)
Divorced or Separated	0	1 (4.3%)	1 (2.4%)
Widowed	0	1 (4.3%)	1 (2.4%)
Employment Status			
Full Time	11 (73.3%)	13 (56.5%)	24 (57.1%)
Part Time	3 (20.0%)	3 (13.0%)	6 (14.3%)
Homemaker	0	1 (4.3%)	1 (2.4%)
Student	0	3 (13.0%)	3 (7.1%)
Disabled	0	1 (4.3%)	1 (2.4%)
Retired	1 (6.7%)	2 (8.7%)	3 (7.1%)
Education Level			
Highschool or Equivalent	0	2 (8.7%)	2 (4.8%)
Some College	3 (20.0%)	5 (21.7%)	8 (19.0%)
Associate Degree	2 (13.3%)	2 (8.7%)	4 (9.5%)
Bachelor’s Degree	6 (40.0%)	6 (26.1%)	12 (28.6%)
Master’s Degree	3 (20.0%)	6 (26.1%)	9 (21.4%)
Doctorate or Equivalent	1 (6.7%)	2 (8.7%)	3 (7.1%)

Note: Frequencies are presented as n (%), descriptives (marked with “*” are presented as M (SD)

## Data Availability

Summarized data is available from the PI upon request. Due to the sensitive nature of the data and small sample size, full data sets and transcripts will not be available.
